# Bandits Who Make Society Their Prey

**Published:** 1898-05

**Authors:** 


### SELECTIONS.

Bandits who Make Society their Prey.—The coachman's bills represent many a dollar never earned; he divides with the tradesmen; unscrupulous veterinary surgeons are parties to the general game for swindling the rich. The fact that there is an organized system of robbery in this city of the majority of its wealthy and fashionable members is not generally known, and the idea would hardly be credited by the intelligent citizen, nor would even the victim of the powerful clique himself believe it possible that he was being held up in such a matter-of-fact and successful manner at first glance. The facts are in evidence, however, and there can be no getting around them. The cheerful victim of this little bunco game is the society man, who feels that he must have his pair of stylish horses, his cob, or his trotter to appear before the public in the proper way. Some there are who go in for big stables, having a new turnout for each entertainment or day in the week. These must pay heavily, indeed, for their little diversion, as the larger the purse the greater amount demanded by the bandits of Manhattan.

The bandits are none other than "Jeems" and "'Enry," the swell coachmen who are in the main smart English horsemen, and who flourish and wax fat on their clever scheme. The coachman is the principal thief, the head of the order of banditti, but he has numerous aids and abettors. The smith who has charge of shoeing, plating, and other work of the kind, is one of his trusty allies; often an unscrupulous veterinarian has a hand in the pie, and altogether the man who finds it impossible to get along in this world without his carriage and pair is fleeced right and left until the wonder is that he will stand it.

Plainly told, the coachman has entire and absolute charge of the horses in almost every stable in this city, and he is permitted to act as he sees fit in the management of the turnout, which means that he has the power to buy horses, have them shod, clipped, fed, and attended to in every way necessary by whoever gives him the largest commission for the privilege of rendering the bills. This is the system, and if anyone believes that the cheerful bandit who has all this power is playing any favorites or overlooking any bets, all he has to do is to get a glimpse at some of the bills that are turned in from the veterinarian, the smith, the feed-man, etc. Some of these are most astounding from a practical point of view, and would naturally indicate that they are never even looked at by the owner who pays them.

There are many tricks of the trade, and they are all worked at some time or other on the unsuspecting society man, who, despite a most stupendous bluff, rarely knows a horse's hock from his withers.

When things go a little slow for "Jeems," "milady" finds it impossible to get her carriage some fine morning, because the horses are lame. This is deplorable, but what are you going to do about it? Simply hire a cab instead. The horses must go to the smith, or the veterinary surgeon must be called to look after them. The next day they are all right, and a comfortable little bill is sent in and paid without question, when it is any odds that there never was anything whatever the matter with them.

The coachman decides who shall attend the stable, and while, of course, he must keep his charges in pretty good general condition, he is not likely to secure the best veterinarian in case of real illness, because the first-class man would hardly enter into any scheme to rob the owner. So the unscrupulous surgeon is called in, charges double the price, and does inferior work. If the owner should by chance have an idea of getting a particular man to do the work, in a majority of cases his plans are knocked in the head by the treachery of the coachman. Medicine ordered is tossed out of the stable window; the horses do not get better; the coachman claims to know a man who can fix them up in no time. He is called, and they are fixed up, and the two conspirators divide the profits. So it goes through all the ways in which it is possible for the horse-owner to be fleeced, beginning with his purchase of the horse, and winding up with the carriage man and the things that are needed about a stable.

The whole system is based, of course, on the ignorance of the owner, and it is surprising that anyone could remain content to know so little about his own property, while so often professing a genuine love for it. It is safe to say that not one out of ten owners of horses in the city has any intelligent idea of them, what it should cost to keep them, when they are sick or well, well or badly fed, shod and cared for, or why he ever bought them in the first place. The whole thing is planned and arranged for him, and he accepts without a question, and pays the bills.

A prominent veterinarian, speaking of the matter to a Sunday *Telegraph* reporter, said: "It is astonishing that the present system is permitted to go on. It is a great evil, and is doing the horse-business a great deal of harm, besides fostering a lot of unscrupulous people who are shrewd enough to take advantage of the utter ignorance of owners on the subject of horses. The main trouble seems to be that no one is brave enough to go to the front and attack it, and also that the average owner would rather be robbed systematically than to take the trouble to find out for himself. I know a number of men who, when told of the state of affairs, replied that they had no time to see that things were right. Others (and these, I believe, are in the majority) have implicit faith in the men in charge of their stables, and leave everything to them. And the stable-men cheerfully take everything that is left to them.—*From the New York Telegraph, Sunday, March* 13, 1898.

